# Research on Non-Contact Monitoring System for Human Physiological Signal and Body Movement

**DOI:** 10.3390/bios9020058

**Published:** 2019-04-19

**Authors:** Qiancheng Liang, Lisheng Xu, Nan Bao, Lin Qi, Jingjing Shi, Yicheng Yang, Yudong Yao

**Affiliations:** 1 School of Sino-Dutch Biomedical and Information Engineering, Northeastern University, Shenyang 110819, China; vin_liangqc@foxmail.com (Q.L.); baonan@bmie.neu.edu.cn (N.B.); qilin@bmie.neu.edu.cn (L.Q.); shijj@bmie.neu.edu.cn (J.S.); y.ang-lin@163.com (Y.Y.); yyao@bmie.neu.edu.cn (Y.Y.); 2 Neusoft Research of Intelligent Healthcare Technology, Co. Ltd., Shenyang 110167, China

**Keywords:** doppler bio-radar, non-contact monitoring system, body movement classify, physiological signals

## Abstract

With the rapid increase in the development of miniaturized sensors and embedded devices for vital signs monitoring, personal physiological signal monitoring devices are becoming popular. However, physiological monitoring devices which are worn on the body normally affect the daily activities of people. This problem can be avoided by using a non-contact measuring device like the Doppler radar system, which is more convenient, is private compared to video monitoring, infrared monitoring and other non-contact methods. Additionally real-time physiological monitoring with the Doppler radar system can also obtain signal changes caused by motion changes. As a result, the Doppler radar system not only obtains the information of respiratory and cardiac signals, but also obtains information about body movement. The relevant RF technology could eliminate some interference from body motion with a small amplitude. However, the motion recognition method can also be used to classify related body motion signals. In this paper, a vital sign and body movement monitoring system worked at 2.4 GHz was proposed. It can measure various physiological signs of the human body in a non-contact manner. The accuracy of the non-contact physiological signal monitoring system was analyzed. First, the working distance of the system was tested. Then, the algorithm of mining collective motion signal was classified, and the accuracy was 88%, which could be further improved in the system. In addition, the mean absolute error values of heart rate and respiratory rate were 0.8 beats/min and 3.5 beats/min, respectively, and the reliability of the system was verified by comparing the respiratory waveforms with the contact equipment at different distances.

## 1. Introduction

Doppler bio-radar has the ability to monitor physiological signals, which depend on the Doppler Effect. Electromagnetic waves can travel through clothing to the surface of the chest cavity and reflect. The antenna receives the reflected waveform containing information on chest displacement. This movement is mainly caused by breathing and the heartbeat [1,2], when the body is still. Otherwise, it contains information about body movement.

Channel State Information (CSI) [3] used a WiFi signal to do research on action recognition [4], indoor localization [5], fall detection [6] and physiological signal detection [7]. Those kinds of studies did not need hardware by using commercial routers and computers. Using self-made devices, Van Dorp [8] judged walking posture from received signals, and Balleri [9] used micro-Doppler signatures to recognize different people by their micro-Doppler signatures.

Self-made devices are also used to monitor a physiological signal, and subjects should also remain stationary as they use the system in the radiation direction of the antenna. In practical use, however, there will be weak signals caused by body movement called Random Body Movement (RBM) in the received signal, even though the subject keeps still. The frequency characteristic of the RBM is complicated, which makes the signal hard to be filtered. The common way to deal with that is to use two identical systems on both sides of the body for compensation. Giovanni [10] monitored users’ heart and respiratory rates as they performed different activities. Li and Lin [11] used arctangent demodulation, and the other way was to use the subject’s location information, which was tracked by amplitude recording to eliminate effects of body movement [12,13]. The existing methods can only work on body movement within a range of 6 cm at a speed of about 0.4 mm/s [14]. In the application of monitoring physical information in real life, however, body movement can be much more strenuous, when they are sitting down or working. In this way, these movements could affect the accuracy of physiological monitoring strongly. When designing the Doppler radar physiological monitoring system in real time, Khan and Ho Cho [15] set two pre-judgment steps to make sure the user was in the effective distance without body movement. They used the auto-correlation width to distinguish between a state of rest and motion. Wenda [16] designed a Doppler radar system for indoor use, which could detect breath and movement of standing, sitting, walking, running and jumping. The breath monitoring could work within 1 m with an accuracy of 87%. The classification accuracy using SVM reached round 85%.

In this article, a non-contact monitoring system for the human physiological signal and body movement was proposed, which was to monitor the health condition of a person who is sitting still for a long time, such as drivers or white-collar workers. Unlike other systems [17], the proposed system worked at 2.4 GHz with only one antenna, since it could send and receive signals in the same time with the transceiver which included transmitter circuit, receiver circuit and others. In addition, the antenna did not only work as a sensor, but also transmitted signals to a serial port module connected with the upper computer. The system could get respiration and heart rates after an action state classification, which could distinguish six kinds of different states.

## 2. System Overview

### 2.1. Hardware System

#### 2.1.1. Antenna

**Figure 1:**
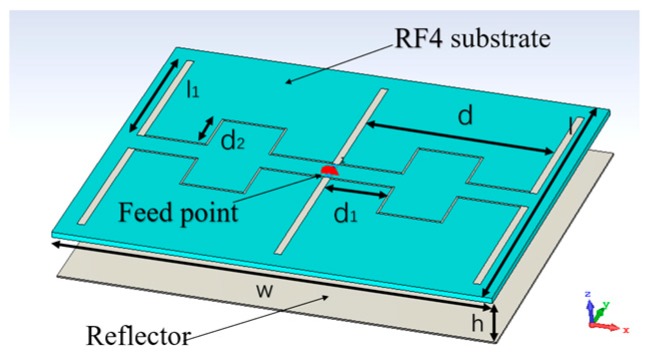
Geometry model of the printed dipole array antenna.

**Figure 2:**
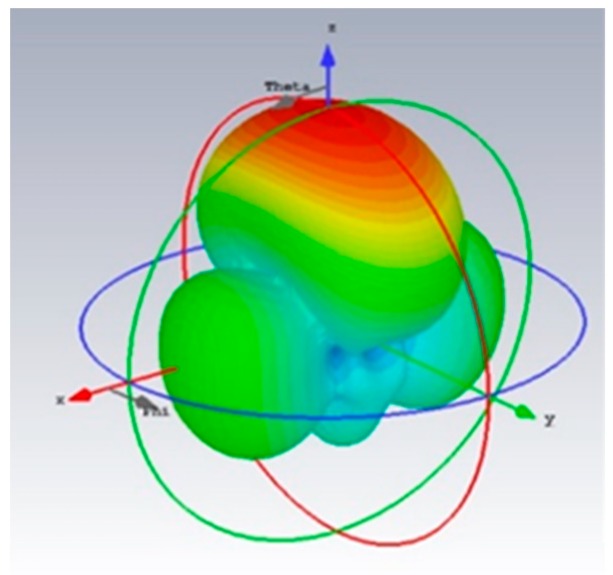
The three-dimensional radiation pattern of the dipole array antenna used in the system.

An antenna plays an important part in a Doppler system, as the performance of the antenna will affect the system. In other words, the application of the Doppler system determines the characteristics of the selected antenna. For monitoring the human physiological signal and body movement, the antenna should not be placed too far from people, however, it does require enough quality of the signal to classify and extract physiological signals [18]. Otherwise, the antenna needs to be focused on only one direction to reduce the influence of other people in the environment. A dipole array antenna was chosen for those demands with a gain of 10.8 dBi and the half-power beam width was 49°. The antenna is shown in Figure 1, and its three-dimensional radiation pattern shown in Figure 2 [19].

#### 2.1.2. Transceiver

**Figure 3:**
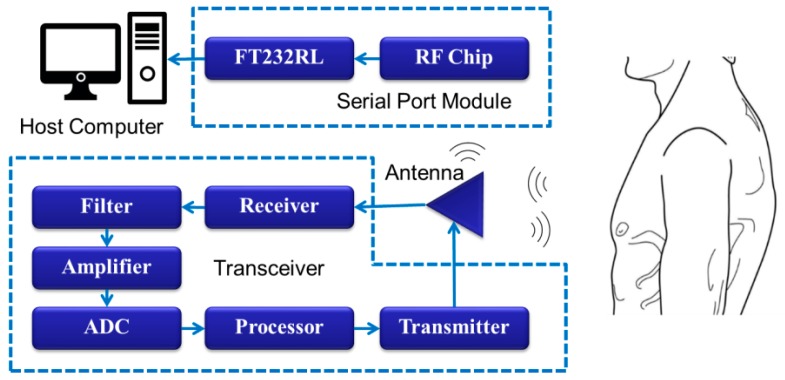
Schematic diagram of hardware system.

**Figure 4:**
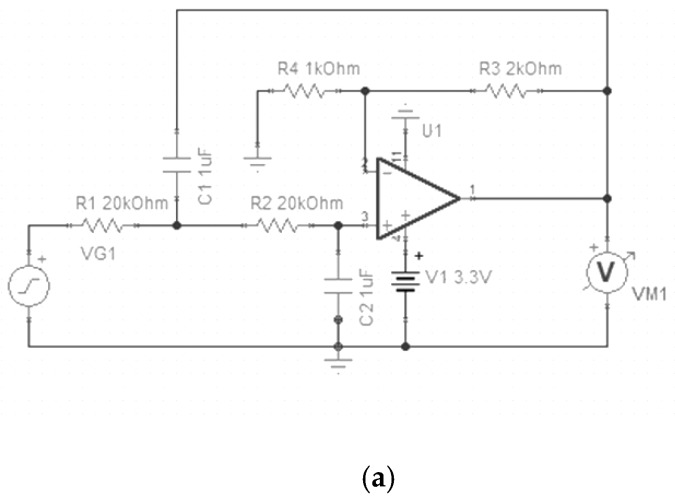

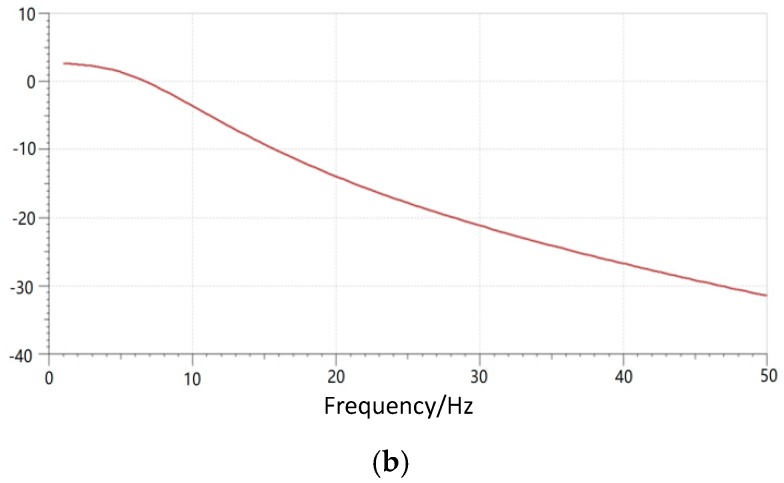
Low pass filter. (**a**) Circuit diagram of low pass filter. (**b**) Bode diagram of low pass filter.

The circuit in the Doppler radar system shown below includes a radio frequency chip, transmitter circuit, receiver circuit and signal processing circuit. The chip handbook provides a circuit diagram of minimum system and Barron circuit. The signal transmission process is shown in Figure 3, after reaching the human body it echoed a signal with 2.4 GHz high frequency pass and a detector tube was used to remove the high frequency noise first. There was a circulator keeping the signal going in the right direction. Since the echo signal which was amplified by amplification circuit contained a low frequency physiological signal and body movement signal together with high frequency noise, a 9 Hz low pass filter was used in the system. The circuit diagram of the filter is shown in Figure 4 with the Gain-Frequency image.

The chip used in the transceiver had several functions. First, it needed to generate a signal at 2.4 GHz. Second, the analogue signal had to be digitized by it. Finally, the digital signal had to be loaded on the continuous wave sent to the human and the serial port module. Thus, nRf24Le01 which worked at 2.4–2.45 GHz was chosen for its 8-bit processor, 1KB in-chip RAM and 14-channel ADC channel up to the 12-bit resolution it had. nRf24Le01 was also cheap as a lower power processor.

#### 2.1.3. Serial Port Module

Previous systems [20] have tended to use cable to connect itself with the upper computer, somehow wasting the transmit ability of the RF signal. Thus, a serial port with radio frequency receiving function was used in this system. It received the digital signal sent from the antenna and passed it to the host computer. This device enabled the system to have a better compatibility with the upper computer though Universal Serial Bus. The digital signal was used by the algorithm in the host computer for classifying and extracting physiological information.

### 2.2. Algorithm Design

#### 2.2.1. Classification Algorithm

To other sensors, signals produced by human activities can be used by pattern recognition helping action recognition. Basic sensing like the acceleration sensor has the ability to reach 90 percent accuracy in distinguishing sleeping, sitting, standing, working and running [21].

**Figure 5:**
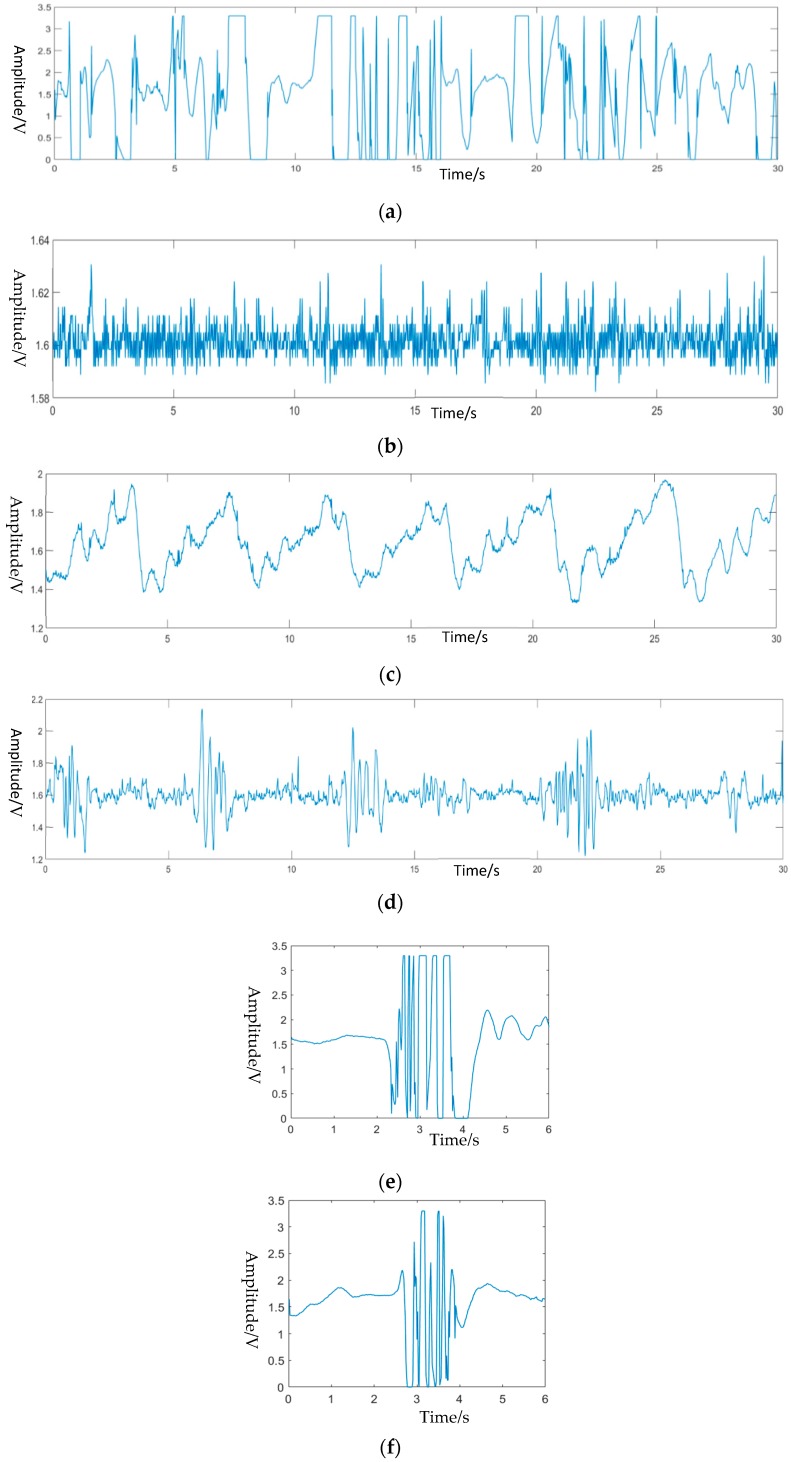
Signals corresponding to different movement states. (**a**) Limb moving. (**b**) Unmanned environment. (**c**) Static measurement. (**d**) A second person entering. (**e**) Sitting down. (**f**) Standing up.

The proposed system focused on removing the influence of large body movement on physiological signal monitoring by classifying them, therefore, the states which were chosen for classification were normal movement or conditions such as: Static measurement (A), limb moving (B), a second person entering (C), unmanned environment (D) standing up (E) and sitting down (F). The signal of these six different movements collected by the system is shown in Figure 5, and the recorded distance change between the antennas to human when they were in a different state. Limb movement, for example, changed the distance between the antenna and participant frequently, which caused dramatic changes in the signal. It also happened when the participant was sitting down or standing up, the drastic fluctuation disappeared when the movement finished. As a second person entered the detection zone, his movement would cause the signal change as well. Only when the participant was sitting without large amplitude body movement, the high-quality signal could be gathered by the system. Additionally, action classification may have helped develop other functions of the system, for example, the time of sitting could also be measured while every instance of “standing up” and “sitting down” was detected.

In the application of monitoring human movement, artificial neural networks [22,23], naive Bayes [24] and support vector machines [25] are the most commonly used. In this paper, two different motion recognition algorithms were designed, which used Support Vector Machine (SVM) and deep learning to classify motion. Among them, when SVM was used, waveform extraction features were selected manually, while when deep learning was used, a bidirectional long and short- term memory network was used for feature extraction of actions. Figure 5 shows the six types of movements that needed to be classified, and there were 300 samples for each movement. The data was five seconds long with a sampling rate of 100 Hz.

The features used in SVM were time duration, maximal upper Doppler shift, maximal low Doppler shift, peak-to-peak bandwidth, mean power of Doppler and standard deviation of Doppler power. Since one SVM classifier could only distinguish between two types, six classifiers were used for classification.

Convolution Neural network (CNN) can make the arbitrary dimension input, but often the input data of the network is two-dimensional. The main idea of CNN is to use the kernel and filter for multi-scale analysis of data, and the data means of this analysis was convolution. Similarly, the image processing algorithm for two-dimensional convolution was used in the neural network. One-dimensional convolution of a one-dimensional signal was to use the convolution function as a filter to filter the signal once and find the characteristics of the waveform at this frequency.

The signal of the six different movements collected by the system is shown in Figure 4, and it recorded the distance change between the antenna and the human when they were in different states. Limb moving, for example, changed the distance between the antenna and participant frequently, which caused dramatic changes in signal. It also happened when the participant was sitting down or standing up, while the drastic fluctuation disappeared when the movement finished. As a second person was entering the detection zone, his movement would cause the signal change as well. Only when the participant was sitting without large amplitude body movement, the high-quality signal could be gathered by the system.

By setting the channel to the number of convolutions, the feature dimension extracted in the convolutional neural network could be changed. The data features in the convolutional neural network appeared in two-dimensional form as a continuous time series, and the other dimension was the time of the sequence. However, in the convolutional neural network, the time series was not taken as continuous signals for information extraction, but each point was taken as a separate unit for feature extraction.

Different from the image information, there was a strong correlation between the adjacent points of the one-dimensional time-domain signal. For convolutional neural networks, the elements are independent of each other, and there is no correlation. Therefore, a special Cyclic Neural Network (RNN) Long Short-Term Memory (LSTM) network became the first choice to deal with the time domain signal problem.

When designing the classification network setting of deep learning, the network model of CNN was used to process the signal at first, because the original signal that was collected contained noise and waveform information of different frequencies. In addition, due to the difference in action duration, different action signals had different characteristics in the time domain. Therefore, a CNN network was required to carry out multi-scale convolution processing on the signal to extract as much as possible the signal information in each frequency and each time domain. In the CNN signal feature extraction part, the algorithm used the vgg-16 model and the network structure between conv1_1 and conv3_3 [26]. In order to ensure the network input format, the network changed to a one-dimensional input. When passing through the three-layer network of the model, 128 different convolution kernels were used for each convolution layer, and 128 convolution times were carried out, and 128-dimensional features were obtained. Then the data was compressed by a pooling layer.

After CNN processing, the signal input bidirectional LSTM was obtained, and the network outputs with 128 channels in each direction. In the actual process of signal processing, the system mainly focused on the peak state of the signal, so a two-way LSTM system was used to ensure that more information was collected. After the LSTM network, a CNN network was needed to adjust the output of the LSTM. The algorithm used three full connection layers to extract semantic features, and the number of channels in their output units was 512, 512 and 6, respectively.

In the process of parameter setting of bidirectional long and short memory network, a batch was set and 64 images were input. The initial learning rate was 0.001. The learning rate was multiplied by 0.1 when 24, 36 and 48 epochs were iterated. SGD was used for the gradient calculation. Two 0.4 drop out values were added between the whole connection layer to prevent over-fitting, and the final output of six values represented the judgment scores of six categories. Soft-max and cross entropy were used as loss functions to conduct the gradient adjustment of the network.

#### 2.2.2. Physiological Signal Algorithm

**Figure 6:**
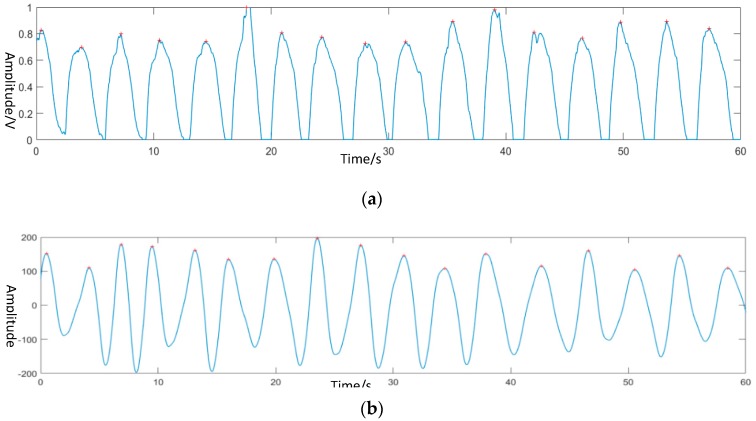
Respiratory signal. (**a**) The original signal from the contact band sensor; (**b**) Reconstructed respiratory signal.

**Figure 7:**
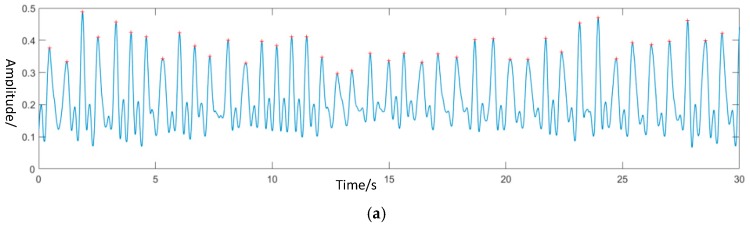

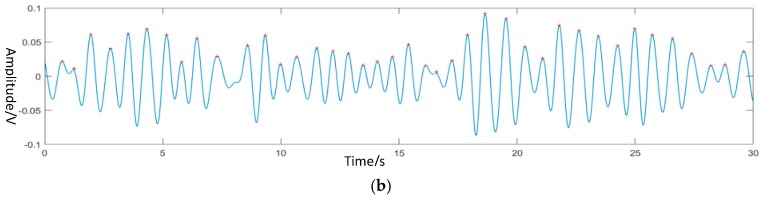
Heartbeat signal. (**a**) The original signal of the pulse sensor. (**b**) Reconstructed body movement signal caused by heartbeat.

After removing the signal from other states, the next step was to extract the physiological signal from state C. The frequency of the respiratory signal and heartbeat signal had its special range, thus, wavelet transform was used to rebuild the signal at a particular frequency and filter noise in other frequency bands. For normal people, the number of breaths and heart beats are 15–20 and 60–100, respectively. Thus, the frequency of respiratory was 0.15 Hz–0.5 Hz and the frequency of the heartbeat was 1 H–2 Hz. The contact devices, which were the pressure sensor for respiratory and heart rate monitoring was used with the non-contact monitoring system together. The waveform of two sensors and the reconstructed respiratory and heart movement signal from the non-contact system is shown in Figure 6 and Figure 7.

## 3. Experiment

**Figure 8:**
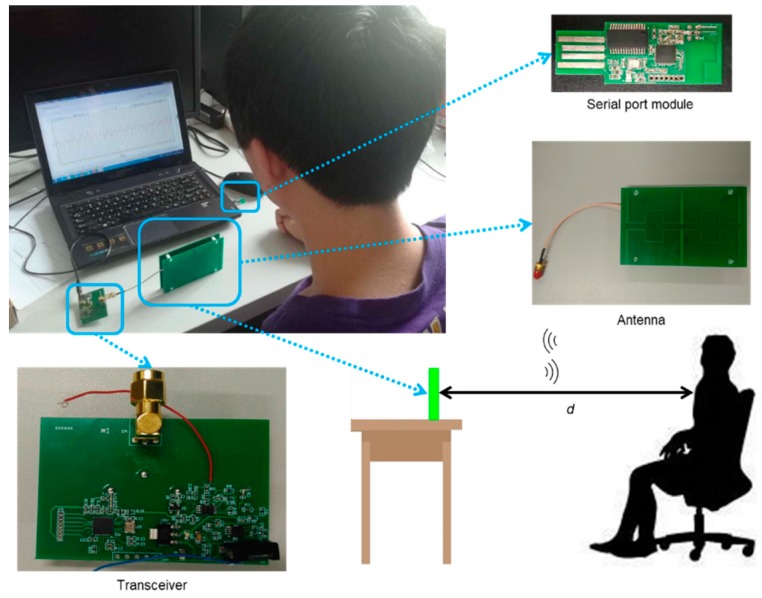
The experiment scene and devices (transceiver, serial port module and antenna).

The experimental scenario is shown in Figure 8. The experimental equipment included contact type breathing monitoring equipment and an electronic sphygmomanometer, pulse sensor and hardware used in the system. In the data collection of motion classification, the system had the ability to classify signals collected from six different situations in Section 2.2. The participator sat in front of the antenna 20 cm, 30 cm and 40 cm to sampling signals (except the “Unmanned environment”). Each of the datapoints was five seconds, and there was 300 data points for every situation.

There were 10 people involved in signal collection, including 6 males and 4 females. During the verification test of cardiac signal and respiratory signal extraction, the pressure sensor of the fingertip was tied to the fingertip of the index finger of the subject to collect pulse data, and the belt-type respiratory sensor was tied to the abdomen diaphragm of the subject to collect breath data.

The three parts of the hardware have been zoomed in on as shown in Figure 8. The two circuits were transceiver and serial port module. A side view of the experiment scene is also shown in Figure 8, and the distance between human and antenna (d) was changed from 20 cm to 100 cm during the experiment. The distance between human and Doppler radar ranged from 20 cm to 80 cm, and two sets of data were measured every 10 cm, with each measurement time of 1 min. A total of 10 people participated in signal collection, including six males and four females. This is because different people breathe in different ways, more testers can improve the accuracy of the system. Attention should be paid to the following aspects in the laboratory:

(1) According to experimental physiology and electromagnetism, it could be known that others will greatly affect the accuracy of the experiment when measuring the equipment. Therefore, the experimental environment with less affected should be a better option.
(2) When the system is turned on and off, it will affect the generated waveform to some extent. In addition, when the system is filtered in the frequency domain, it will also affect the waveform at the edge. Therefore, during the verification test of cardiac signal and respiratory signal extraction, more than one minute of data should be collected in the experiment, and the middle one minute of data should be used for calculation, so as to avoid the influence of the above phenomenon on the experimental results.
(3) Try to ensure the diversity of samples when collecting classified actions, that is, each action has data collected by 10 subjects.
(4) In the verification test of cardiac signal and respiratory signal extraction, try to ensure that the range of each breath of the subject is the same. This verification can be generally judged by the waveform form collected by the contact device.

## 4. Results and Discussion

### 4.1. Effective Distance

As a non-contact device for monitoring a physical signal, it was imperative to design an experiment for distance testing, where the performance of each distance could be judged by the energy of the signal. The experiment involved ten participants, including six males and four females. A fingertip pressure sensor and a breath sensor were placed on the participant’s body together with an antenna which was a part of the Doppler radar system located in front of the participant. The length range between them was 20 cm to 80 cm with seven equidistant collection points. The measurement time was 1 min for each experience, and every participant needed to do two experiments in one point.

After extracting the waveform of breath and heartbeat, the energy of the original signal, the breath signal and the heartbeat signal were calculated by the formula below:

(1) $$E=\overset{n}{\underset{i=1}{\sum }}S{\left(i\right)}^{2}$$

**Figure 9:**
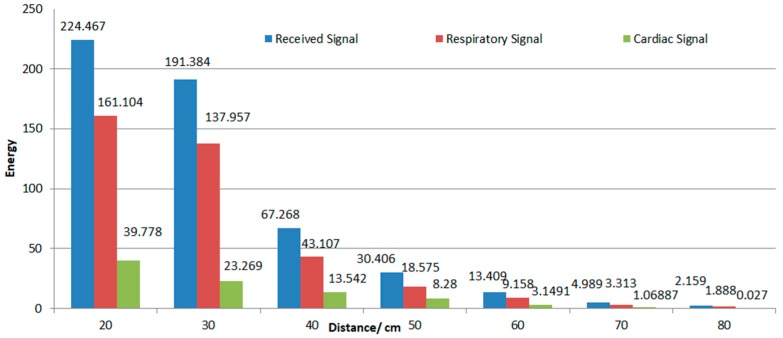
The relation between electromagnetic wave energy and distance received by antenna.

The histogram diagram of energy and distance was made according to the average energy of 20 1-min antenna receiving waveforms collected at each position from 20 cm to 80 cm. At the same time, the respiration rate and the heart rate were extracted, and the energy was calculated and is shown in Figure 9. It can be seen that the energy received by the antenna dropped suddenly at 40 cm and almost disappeared at 60 cm. However, the energy of the cardiac signal basically disappeared at 50 cm. Therefore, the maximum normal working distance of this antenna was about 40 cm.

### 4.2. Action Classification

When the SVM carried out 100 iterations, the accuracy was stable at around 80.72%. The confusion matrix at this time is shown in Table 1. When using the deep learning network to classify six different types of motion, a 4-fold cross-validation method was selected to verify the accuracy of the algorithm. The idea of folded cross validations was to divide all the data into four equal parts and use one of them as a test set to calculate the accuracy of the model, and the other three as a training set to train the model. After adjusting the learning rate and the loss function in the training, the confusion matrix of the classification calculation algorithm shown in Table 2 could be obtained.

It can be seen from Table 2 that the accuracy reached 88% when the deep learning network was used to classify the states of movement. In addition to the low degree of differentiation between the sudden changes of standing and sitting, the rest of the states could be basically judged accurately and the misjudgment of standing and sitting often occurred in mutual discrimination. In this case, a simple method to improve the accuracy of the algorithm was completed, that is, by judging the movements before and after the standing and sitting of the algorithm, so as to correctly distinguish the standing and sitting.

**Figure 10:**
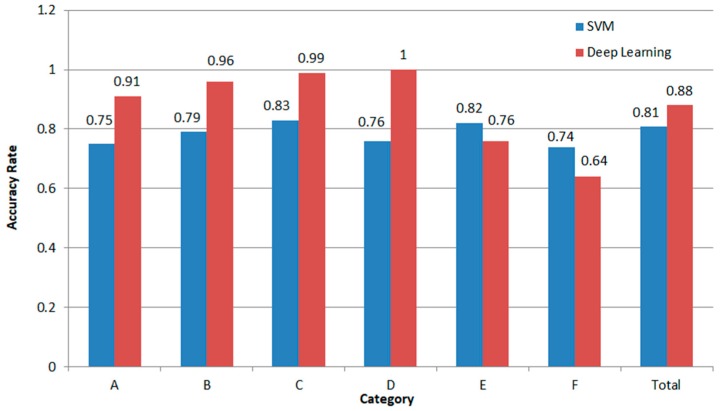
Accuracy comparison of deep learning algorithm and Support Vector Machine algorithm in RF signal state classification.

Figure 10 shows the comparison of the total accuracy of the two classification methods and the accuracy of the six somatic classification methods. It can be seen that the classification method based on SVM had an average accuracy of judgment for each action, indicating that the features extracted manually mainly focused on the morphological features of waveform. The total accuracy of the deep learning algorithm was relatively high, and the main reason that it affected the accuracy was that it made more errors in the judgment of the interaction between body movements with a sudden change in the states of standing and sitting. The accuracy of classifying them with other actions was 96.0% showing in Table 2. One way to improve the classification algorithm is to use it in the real time system which can record human’s previous movement state. For example, if there had already existed a “standing up” the next “standing up” should have been “sitting down.”

Therefore, it can be concluded that the motion classification algorithm of deep learning is very effective for the accurate judgment of physiological signals and the monitoring and classification of signals of body movement.

### 4.3. Physiological Signal Extraction

The absolute error in the number of the cycles between the reference signal and the reconstructed signal within one minute was selected to measure the extracted accurate value, and the absolute error in average time between the peaks within one minute, which could also be used as the absolute index of the average accurate difference.

**Figure 11:**
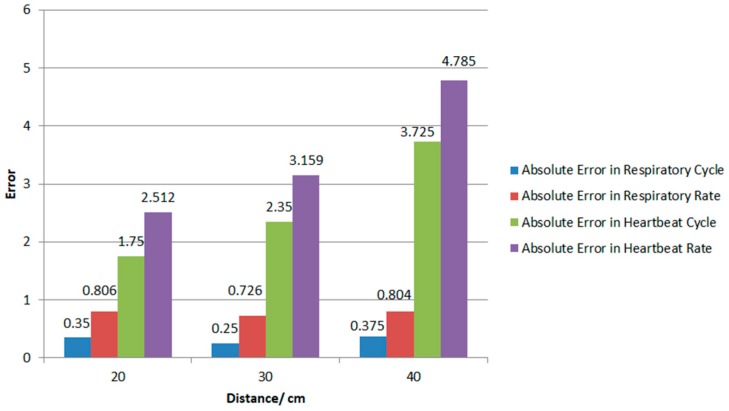
Error analysis of three different distances.

Figure 11 shows the number of cycles extracted from 20 one-minute data at 20 cm, 30 cm and 40 cm in each position and the mean value of the absolute error of the average heart rate and respiration rate of the Doppler radar system that was simultaneously monitored using the contact heart rate and respiration measuring equipment as the control group. As shown in the figure, the accuracy of the test decreased with an increase in the distance. The monitoring of respiration rate was more accurate than the monitoring of heart rate at the same distance. Moreover, the accuracy of heart rate monitoring had a larger error with the change of distance. According to the previous analysis, this is caused by the decrease of waveform energy caused by distance and the low energy of the received cardiac signal.

## 5. Conclusions

The Doppler radar system proposed in this paper is suitable for classifying six different body movement signals with the classification method of deep learning and can also monitor physiological signals in a distance which is less than 40 cm. The accuracy of the classifier was 88% and could be further improved in the system. While the respiratory waveform, cardiac waveform and contact devices were compared at 20 cm, 30 cm and 40 cm respectively, and the errors of the respiratory cycle number, average respiratory rate, cardiac cycle number and average heart rate at the corresponding positions were obtained. Compared with the contact device, the average error of respiration rate in the optimal measurement distance was about 0.8 beats/min, and the average error of the heart rate was about 3.5 beats/min, which verified the accuracy of the algorithm. Compared with the existing system, this system has a better performance in the classification of action state which is shown in Table 3. What’s more, it has the ability to monitor the heartbeat. However, the effective distance for the system is shorter than the previous one [16], this feature makes it more suitable in offices or cars.

## Figures and Tables

**Table 1:** Support Vector Machine classification accuracy after genetic selection algorithm optimization.

	A	B	C	D	E	F
A	0.75	0.04	0.03	0.12	0.02	0.06
B	0.11	0.79	0.02	0.07	0.03	0.05
C	0.02	0.04	0.83	0.04	0.01	0.04
D	0.09	0.04	0.03	0.76	0	0.01
E	0.01	0.06	0.05	0.01	0.82	0.10
F	0.02	0.03	0.04	0	0.12	0.74

**Table 2:** Classification accuracy of the deep learning algorithm.

	A	B	C	D	E	F
A	0.91	0	0.01	0	0	0.04
B	0.01	0.96	0	0	0.01	0
C	0.08	0.02	0.99	0	0.01	0.01
D	0	0	0	1	0	0.01
E	0	0.02	0	0	0.76	0.30
F	0	0	0	0	0.22	0.64

**Table 3:** The comparison between two systems.

	[16]	Proposed System
Maximum distance	1 m	0.4 m
Methods of classification	SVM	VGG-16 and LSTM
Classification accuracy	85%	>88%
Error of respiratory detection	13%	0.779 beats/min
Error of Heart rate detection	No experiment	3.49 beats/min
